# The use of indicators for the management of Mental Health Services

**DOI:** 10.1590/1518-8345.4202.3409

**Published:** 2021-04-12

**Authors:** Inacia Bezerra de Lima, Filipe Andrade Bernadi, Diego Bettiol Yamada, Andre Luiz Teixeira Vinci, Rui Pedro Charters Lopes Rijo, Domingos Alves, Antonia Regina Ferreira Furegato

**Affiliations:** 1 Universidade de São Paulo, Escola de Enfermagem de Ribeirão Preto, PAHO/WHO Collaborating Centre for Nursing Research Development, Ribeirão Preto, SP, Brazil.; 2 Scholarship holder at the Coordenação de Aperfeiçoamento de Pessoal de Nível Superior (CAPES), Brazil.; 3 Universidade de São Paulo, São Carlos, SP, Brazil.; 4 Universidade de São Paulo, Faculdade de Medicina de Ribeirão Preto, Ribeirão Preto, SP, Brazil.; 5 Instituto Politécnico de Leiria, Escola de Tecnologia e Gestão, Leiria, Leiria, Portugal.

**Keywords:** Health Status Indicators, Public Health Administration, Mental Health Services, Health Services Administration, Health Care Quality Indicators, Health Planning Guidelines, Indicadores Básicos de Saúde, Administração em Saúde Pública, Serviços de Saúde Mental, Administração de Serviços de Saúde, Indicadores de Qualidade em Assistência à Saúde, Diretrizes para o Planejamento em Saúde, Indicadores de Salud, Administración en Salud Pública, Servicios de Salud Mental, Administración de los Servicios de Salud, Indicadores de Calidad de la Atención de Salud, Directrices para la Planificación en Salud

## Abstract

**Objective:**

to identify indicators that can be used in the management of Mental Health Services.

**Method:**

an integrative review in which we adopted the Population, Concept, and Context strategy to formulate the following Guiding Question: “Which indicators can be used for the management of mental health services?”.

**Results:**

a total of 22 articles were included and divided into two main groups: countries with initial high income (54%) as well as low- and middle-income countries (46%). We identified 5 studies that had experienced the use of indicators, 5 studies that had reported partial implementation, 9 studies that did not report use or implementation, 1 study on the indicator selection process, 1 as an implementation pilot, and a final study with a discussion for implementation. High-income countries also find it difficult to implement mental health indicators. The main difficulties in adopting the use of indicators are lack of basic mental health services, financial resources, legislation, political interest, and guidelines for its management.

**Conclusion:**

it is unusual to find a descriptive comparison of quality monitoring programs at the system level in the technical-scientific literature related to mental health indicators.

## Abstract (pt)

**Objetivo:**

identificar indicadores que possam ser utilizados na gestão dos Serviços de Saúde Mental.

**Método:**

revisão integrativa em que adotou-se a estratégia População, Conceito e Contexto para formular a seguinte questão norteadora: “Quais indicadores podem ser usados para a gestão dos serviços de saúde mental?”.

**Resultados:**

um total de 22 artigos foram incluídos e divididos em dois grupos principais: países com renda inicial alta (54%), bem como países de baixa e média renda (46%). Identificamos 5 estudos que experimentaram o uso de indicadores, 5 estudos que relataram implementação parcial, 9 estudos que não relataram uso ou implementação, 1 estudo sobre o processo de seleção de indicadores, 1 como piloto de implementação e um estudo final com uma discussão para implementação. Os países de alta renda também têm dificuldade para implementar indicadores de saúde mental. As principais dificuldades na adoção do uso de indicadores são a falta de serviços básicos de saúde mental, recursos financeiros, legislação, interesse político e diretrizes para sua gestão.

**Conclusão:**

é incomum encontrar uma comparação descritiva de programas de monitoramento de qualidade no nível de sistema na literatura técnico-científica relacionada a indicadores de saúde mental.

## Abstract (es)

**Objetivo:**

identificar indicadores que se puedan utilizar en la gestión de Servicios de Salud Mental.

**Método:**

revisión integradora en la que adoptamos la estrategia Población, Concepto y Contexto para formular la siguiente Pregunta Orientadora: “¿Qué indicadores se pueden utilizar para la gestión de servicios de salud mental?”.

**Resultados:**

se incluyó un total de 22 artículos y se los dividió en dos grupos principales: países con ingresos altos iniciales (54%) y países con ingresos bajos y medios (46%). Identificamos 5 estudios que habían experimentado el uso de indicadores, 5 estudios que habían reportado implementación parcial, 9 estudios que no reportaron uso o implementación, 1 estudio sobre el proceso de selección de indicadores, 1 como piloto de implementación y un estudio final con una discusión para la implementación. Los países de ingresos altos también tienen dificultades para implementar indicadores de salud mental. Las principales dificultades para adoptar el uso de indicadores son la falta de servicios básicos de salud mental, recursos económicos, legislación, interés político y directrices para su gestión.

**Conclusión:**

es inusual encontrar una comparación descriptiva de los programas de monitoreo de la calidad a nivel de sistema en la literatura técnico-científica relacionada con indicadores de salud mental.

## Introduction

The World Health Organization (WHO) has set four priority objectives in its Mental Health Action Plan 2013-2020^(^ 1 ^)^: strengthen effective leadership and governance for Mental Health (MH); provide comprehensive, integrated and responsive mental and social health services in community settings; implement strategies for promotion and prevention in MH; and strengthen information systems, evidence and research for MH^(^ 1 ^-^ 2 ^)^. One of the principles for achieving these WHO goals is the use of indicators that are important for monitoring MH systems data. The WHO recommends that 80% of all countries collect and report at least one core set of Mental Health Indicators (MHIs) and that this action should happen through their national health and social information systems by the year 2020^(^ 3 ^)^. Moreover, it also provides a set of key indicators to assess the levels of implementation, progress, and impact of defined targets. After the publication of the action plan, the WHO launched the Mental Health Atlas of 2014 and 2017 to monitor the progress of countries in achieving the established targets^(^ 4 ^-^ 5 ^)^.

More than 450 million people are afflicted by mental illness and the global burden of mental illness is underestimated. Recent research suggests that this burden accounts for 32.4% of years lived with disability and 13.0% of disability-adjusted life-years. This is a particular concern in low- and middle-income *countries* (LMICs) where more than 70% of the mental illnesses occur^(^ 6 ^)^.

In the last two decades, there have been a large number of publications and reviews on the use of MH guidelines^(^ 7 ^-^ 8 ^)^. Despite the proliferation of evidence-based guidelines for the treatment of mental disorders, there is no consensus as to which recommendations should be used^(^ 9 ^)^. A set of indicators should follow expected patterns of use, along with relevant and necessary data, in addition to validity accuracy to inform the merits of the evaluated practices and processes^(^ 10 ^)^. There were a limited number of “evaluative indicators” in MH related findings to record or measure properties, process, and interpretation of use and outcomes^(^ 10 ^-^ 11 ^)^.

This scenario shows the lack of focus on this aspect prior to the publication of the WHO’s Mental Health Action Plan from 2013 to 2020, and that it is now necessary to know the possible progress derived from the action based on such a document.

In spite of the WHO’s recommendations, it is possible to find in the literature differences in the groups of indicators, the name of the indicators, how they are defined and which category each one belongs to^(^ 1 ^,^ 12 ^)^. Thus, it is important to seek evidence on the indicator’s performance for MH management based on the experience of use analysis, highlighting the differences and consensus of interpretations. In this way, we will carry out an integrative review of the technical-scientific literature, with the main objective of identifying indicators that can be used for the management of MH services. In this study, we will also analyze the evolution of MH services in different contexts and countries, the development of indicators and the progress of their implementation. Finally, it is important to highlight that this study is part of an international multicenter study, involving researchers from Brazil and Portugal.

## Method

This Integrative Review study was prepared according to the method described in the Joanna Briggs Institute Reviewers’ Manual 2015 - JBISRIR^(^ 13 ^-^ 14 ^)^. The study mapped the main concepts, allowing for the clarification areas of research and identifying knowledge gaps. This can be done through an assessment of feasibility, significance, and adequacy of the recommended health care practice. In our scenario, this is fundamental to understand the evolution and state of the art of MHI services in different contexts and countries.

This Integrative Review is, however, not in itself a normative contribution. It does not aim to propose or argue for what core indicators and values should guide MHIs, though the importance of such work is emphasized. Instead, the Integrative Review has a descriptive and analytic function. It seeks to provide an overview of what issues have emerged on MHIs and what guidance exists on how to address them and inform ongoing efforts to develop meaningful and comprehensive guidelines for the practice.

A structure of six topics was proposed: (1) Identifying the research guiding question (GQ); (2) Identifying relevant studies; (3) Selecting studies; (4) Data selection and storage; (5) Collating, summarizing and reporting results; and (6) Results Analysis - Discussion^(^ 15 ^)^.

*1: Identifying the research GQ.* To fulfill the requirements of the integrative review, the research GQ must adequately establish the fundamental evidence for the argument in the GQ. It should also determine the incorporation of the analysis, promoting the amount of information in the databases, with fewer unnecessary searches^(^ 13 ^)^.

To construct the research GQ, we adopted the Population, Concept and Context (PCC) strategy. With this strategy, in this study, we formulated the following GQ: “Which indicators can be used for the management of MH services?”, where Population refers to the MHIs, Concept refers to the use in management, and Context refers to the MH services.

It should be emphasized that the population may include the articles selected for inclusion and must be related to the objectives of the integrative review. The Concept should be clearly articulated for the integrative character and breadth of the survey. The Context should be clearly defined and can include considerations of cultural factors, such as geographic location and/or specific racial or gender interests. In some cases, the context may also cover details about specific scenarios such as the health care system^(^ 15 ^)^.

The framework suggests a broad, clearly articulated research GQ, defining concepts, target population, health outcomes, and integrative, whilst also accounting for the aim and rationale of the review^(^ 15 ^)^.

*2: Identifying relevant studies.* A search was performed in the following databases: Web of Science, National Library of Medicine - PubMed, Science Direct, MEDLINE and Scopus. Furthermore, additional searches were made in databases with the majority of papers being in the Portuguese and Spanish languages, namely, Scientific Electronic Library Online (SciELO) and Latin-American and Caribbean System on Health Sciences Information (LILACS). In these searches, we used the selected keywords and a translation of the selected descriptors by the Health Sciences Descriptors^(^ 16 ^)^.

*3: Selecting studies.* The search procedure was oriented in accordance with the combination of keywords derived from the PCC strategy of this study and controlled and uncontrolled descriptors from Medical Subject Headings - MESH, a dictionary of controlled vocabulary synonyms for indexing articles^(^ 16 ^)^. For the combination of such terms, we considered the Boolean operators AND, OR and NOT to compose the search queries in the referred databases, following the inclusion and exclusion criteria. Within the PCC strategy, the search controlled descriptors were the following: Population (P): “Quality Indicators, Health Care” OR “Health Status Indicators” OR “Health Planning Guidelines”. Uncontrolled descriptors for Population: “Quality Indicators, Healthcare” OR “Healthcare Quality Indicator” OR “Healthcare Quality Indicators” OR “Indicator, Healthcare Quality” OR “Indicators, Healthcare Quality” OR “Quality Indicator, Healthcare” OR “Global Trigger Tool, Healthcare” OR “Healthcare Global Trigger Tool” OR “Health Status Indicator” OR “Indicator, Health Status” OR “Indicators, Health Status” OR “Health Status Index” OR “Health Status Indices” OR “Index, Health Status” OR “Indices, Health Status” OR “Health Status Indexes” OR “Indexes, Health Status” OR “Health Risk Appraisal” OR “Appraisal, Health Risk” OR “Appraisals, Health Risk” OR “Health Risk Appraisals” OR “Risk Appraisal, Health” OR “Risk Appraisals, Health” OR “Guideline, Health Planning” OR “Guidelines, Health Planning” OR “Health Planning Guideline” OR “Planning Guideline, Health” OR “Planning Guidelines, Health” OR “Guidelines for Health Planning. Controlled descriptors for Concept (C): “Health Facility Administration” OR “Public Health Administration” OR “Hospital Administration” OR “Health Services Administration”. Uncontrolled descriptors for Concept: “Administration, Health Facility” OR “Facility Administration, Health” OR “Administration, Public Health” OR “Administration, Hospital” OR “Hospital Organization and Administration” OR “Organization and Administration, Hospital” OR “Administration, Health Services”. Controlled descriptors for Context(C): “Mental Health” OR “Mental Health Services”. Uncontrolled descriptors for Context:- “Health Services, Mental” OR “Health Service, Mental” OR “Mental Health Service” OR “Service, Mental Health” OR “Services, Mental Health”.

The inclusion criteria for the articles retrieved from the database search were as follows: publications in languages mastered by the authors (English, Spanish and Portuguese); publications in the last 15 years (2003-2018) that were determined by their compatibility with the studies used in the WHO Mental Health Action Plan 2013-2020^(^ 1 ^)^; published articles; qualitative and quantitative studies; the search sources may include any existing literature, namely primary research studies, systematic reviews, meta-analyses, studies linked to countries that have a public health system, and that the search be left as “open”, allowing for the inclusion of all documents and justifications.

The exclusion criteria were given as follows: studies out of the desired integrative review; articles and documents that were not available in an electronic format or could not be accessed due to payment restrictions; articles related to countries that do not have a public health system; indicators of other health areas, websites, and advertisements in the media.

*4: Data selection and storage.* The organization of this process was made using two software programs, Mendeley (https://www.mendeley.com) and Rayyan (https://rayyan.qcri.org/), to manage and share research documents.

*5: Collating, summarizing and reporting results.* This step was the elaboration of a narrative synthesis describing the objectives and the purposes of the selected and reviewed documents, the concepts adopted and the results related to the issue of this review^(^ 17 ^)^.

*6: Results Analysis - Discussion.* Finally, the articles obtained were analyzed between high-income countries (HICs) and LMICs as well as how they make use of MHIs in the management of services, financial resources and policies for the creation and implementation of MHIs.

This study is part of a larger international multicenter project involving researchers from the Ribeirão Preto Medical School and the Ribeirão Preto School of Nursing in Brazil, in partnership with the Polytechnic Institute of Leiria, Portugal. Thus, it is important to highlight that the steps described above were made with the participation of these researchers.

## Results

We executed a structured search with defined strategies in the respective database platforms, along with controlled descriptors, uncontrolled descriptors, and keywords. It resulted in 929 papers among all databases and, after a duplicity analysis, 125 papers were removed. Then, using the procedures defined and refined from the inclusion and exclusion criteria combined with this study’s GQ, an initial screening of the papers’ titles and abstracts resulted in an exclusion of 804 records. Afterward, a full-text reading was performed on the papers left aiming to identify articles that addressed the GQ of this study. As a result of this step, a total of 22 articles were selected to be part of the Integrative Review. The selection criteria applied in this Integrative Review were performed by two researchers and submitted to a third one for review.

In order to organize reports and present systematic results according to the proposed approach, we used the Preferred Reporting Items for Systematic Reviews and Meta-Analyses (PRISMA). The screening of the selected studies in this study was summarized in the 4-step prism flowchart: identification, screening, eligibility, and included^(^ 18 ^)^.

**Figure 1:**
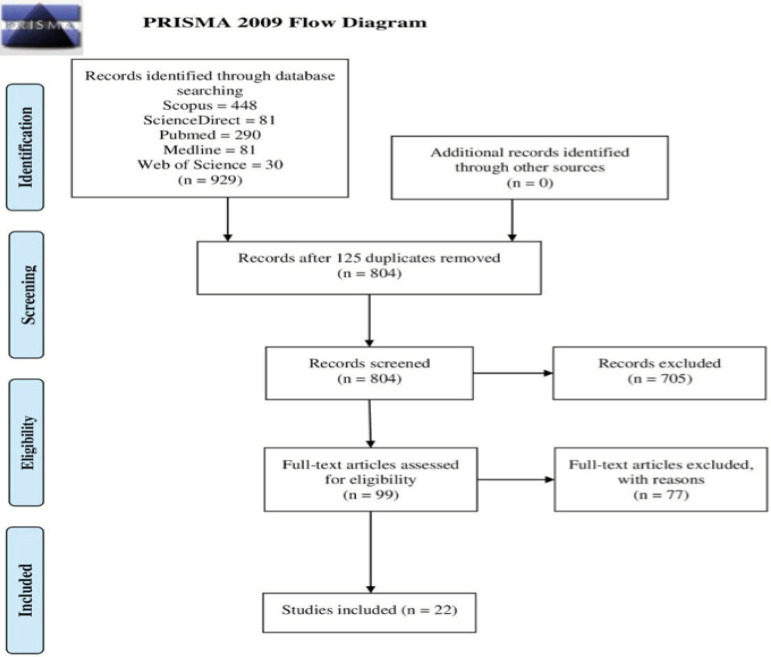
PRISMA 2009 Flow Diagram adapted^(^ 17 ^)^

A brief presentation of this process and its characteristics can be seen in the PRISMA flow chart presented in Figure 1.

We present the analysis of the 22 studies identified in the database search. Initially, we divided the findings into two main groups: HICs (54%) and LMICs (46%). Also, the studies were organized in chronological order. In the first group, we present studies conducted for HICs, while the main characteristics of the second group are LMICs. Then, each group was classified into conforming subgroups in characteristic of the population studied, i.e., studies involving a group of countries, countries or regions of countries were categorized. Next, we showed a more detailed analysis of the studies found, following the previous division described, in accordance with their income level.

Considering the studies in the main group of HICs, we identified 5 papers related to the group of countries, the papers belonging to the WHO, the Organization for Economic Cooperation and Development (OECD), the Statistical Office of the European Communities (Eurostat) and the Intercontinental Marketing Services (IMS)^(^ 19 ^-^ 23 ^)^, as shown in Figure 2 below.

**Figure 2:** High-income regions

Study	Study Objective	Territorial dimension	Implementation of indicators
Psychosocial well-being and psychiatric care in the European Communities: analysis of macro indicators(19)	To review macro indicators capable of providing a synthetic description of mental health status and the availability of psychiatric care in European countries	Europe (OECD, EUROSTAT^‡^ and IMS^§^)	The authors do not report the experience of using the indicators in the management
New perspectives of mental health service(20)	The study searches methodologies for the use of health indicators, based on characteristics of the users of health services	Italy, Austria, Estonia, Finland, France, Italy, Spain, Norway, Romania and UK (England, Scotland, Wales and Northern Ireland)	The authors do not report the experience of using the indicators in the management
Monitoring of mental health care at the system level: country profiles and EU* country status(21)	To provide a descriptive overview of Quality Monitoring Programs status in European countries	England, Denmark, France, Germany, Italy, Netherlands, Portugal, and Sweden	Partial implementation
Reporting and use of OECD^†^ Quality Indicators for Health Care at the national and regional levels in 15 countries(22)	To explore reports on the use of quality indicators in OECD member countries	Belgium, Canada, Czech Republic, France, Germany, Ireland, Israel, Latvia, Netherlands, Norway, New Zealand, Slovakia, Sweden, United States and UK (England, Scotland, Wales and Northern Ireland)	Partial implementation
UK Quality Indicator Project^®^ (UK QIP) and the UK (England, Scotland, Wales and Northern Ireland) independent health care sector: a new development(23)	To describe the implementation of the UK Quality Indicators Project in the health sector	UK (England, Scotland, Wales and Northern Ireland)	Implementation in use

* EU = European Union;

† OCDE = Organization for Economic Cooperation and Development;

‡ EUROSTAT = Statistical Office of the European Communities;

§ IMS = Intercontinental Marketing Services

The remaining studies consisted of 6 related to HICs^(^ 24 ^-^ 29 ^)^and one related to a high-income specific region^(^ 30 ^)^, as shown in Figure 3 below.

**Figure 3:** High-income countries

Study	Study Objective	Territorial dimension	Implementation of indicators
Overview of the healthcare system in the Czech Republic^(^ 24 ^)^	To describe the Czech mental health system through population indicators	Czech Republic	The authors do not report the experience of using the indicators in the management
Development of Mental Health Indicators in Korea^(^ 25 ^)^	To develop ways to measure the state of mental health in Korea by analyzing indicators in other regions	Korea South	Indicators were only selected
Call for information, call for quality in mental health care^(^ 26 ^)^	To build a model to improve the quality of mental health services mediation system at regional and local levels	Italy	The authors do not report the experience of using the indicators in the management
Size Matters - Determinants of Modern, Community-Oriented Mental Health Services^(^ 27 ^)^	To explore the quality and quantity of substance abuse-related mental health services, and evaluate the correlation between the needs of the population and the availability of those services	Finland	Implementation in use
Quality indicators for the referral process from primary to specialized mental health care: an explorative study in accordance with the RAND* appropriateness method^(^ 28 ^)^	To develop quality indicators to detect the impact of quality of primary care referral information to specialized mental health care has on the quality of mental health services	Norway	The authors do not report the experience of using the indicators in the management
Mental health quality, outcome measurement, and improvement in Germany^(^ 29 ^)^	To describe the most recent results of quality assurance programs for mental health services in Germany	Germany	Implementation in use
Composing a Core Set of Performance Indicators for Public Mental Health Care: A Modified Delphi Procedure^(^ 30 ^)^	To describe the development of a set of performance indicators that are feasible, meaningful and useful for assessing the quality of the public mental health system in Amsterdam	Netherlands	Pilot implementation

* RAND = The RAND/UCLA Appropriateness Method (RAM) was developed in the mid-1980s, as part of the RANDCorporation/University of California Los Angeles (UCLA) Health Services Utilization Study, primarily as an instrument to enable the measurement of the overuse and underuse of medical and surgical procedures

For the other group of selected studies, we identified 7 articles related to groups of LMICs^(^ 31 ^-^ 37 ^)^, as shown in Figure 4 below.

**Figure 4:** Low-income and middle-income regions

Study	Study Objective	Territorial dimension	Implementation of indicators
Financing mental health services in low- and middle-income countries^(^ 31 ^)^	To evaluate the impact of health care financing deals on the efficient and equitable use of mental health services	Azerbaijan, Bulgaria, Georgia, Lithuania, Kyrgyzstan, Pakistan, Nepal, Thailand, Malaysia, Chile, Kenya, Zambia	The authors do not report the experience of using the indicators in the management
Scale up services for mental disorders: a call for action^(^ 32 ^)^	To improve mental health services through the search for financing and monitor this improvement through indicators	Chile, Albania, Ethiopia, Thailand, China (Hunan Province), Iran, Nepal, Morocco, Nigeria, Ukraine, Vietnam, Paraguay	Partial implementation
Mental health systems in countries: where are we now?^(^ 33 ^)^	To analyze ways to improve health systems in low- and middle-income countries	Brazil, India and South Africa	Partial implementation
Three models of community mental health services in low-income countries^(^ 34 ^)^	To compare three models of community mental health services in low-income settings	Nigeria, Philippines, and India	The authors do not report the experience of using the indicators in the management
Situational analysis: preliminary regional review of the Mental Health Atlas 2014^(^ 35 ^)^	To consolidate the data provided for the Atlas 2014 questionnaire by the Member States of the Eastern Mediterranean region	22 Member States of the Eastern Mediterranean Region	Partial implementation
Indicators for routine monitoring of effective mental health coverage in Low- and Middle-Income Countries (LMIC) environments: a Delphi study^(^ 36 ^)^	To identify indicators for the measurement of effective coverage of mental health treatment through a Delphi Study	Ethiopia, India, Nepal, Nigeria, South Africa and Uganda	The authors do not report the experience of using the indicators in the management
Evaluating capacity-building for mental health system strengthening in low- and middle-income countries for service users and caregivers, service planners and researchers^(^ 37 ^)^	To evaluate the impact of human resources training in low- and middle-income countries	Ethiopia, India, Nepal, Nigeria, South Africa and Uganda	The authors do not report the experience of using the indicators in the management

We also identified one study that describes LMICs individually^(^ 38 ^)^, while the other two refer to specific regions of LMICs^(^ 39 ^-^ 40 ^)^. As shown in Figure 5 below:

**Figure 5:** Low-income and middle-income countries

Study	Study Objective	Territorial dimension	Implementation of indicators
Public sector mental health systems in South Africa: Inter-provincial comparisons and policy implications(38)	To document current levels of provision of public health mental health services in South Africa and to compare services between provinces	South Africa	Implementation in use
Evaluation of results and impact of the first phase of a community based mental health model in localities in Bogotá, D.C.(39)	To evaluate the impact of the Community Based Mental Health Model through indicators	Colombia	Implementation in use
Development of mental health indicators at the district level in Madhya Pradesh, India: mixed methods study(40)	To develop a basic set of indicators to monitor mental health in primary care settings through a Mixed Methods Study	India	Implementation in discussion

According to the subject matter presented, we identified that 5 studies effectively reported the experience of using MHIs and 3 of them described high-income environments^(^ 23 ^,^ 27 ^,^ 29 ^,^ 38 ^-^ 39 ^)^. Another 5 studies reported partial implementation of MHIs^(^ 21 ^-^ 22 ^,^ 32 ^-^ 33 ^,^ 35 ^)^. An additional 9 papers didn’t report the use or implementation of developed indicators^(^ 19 ^-^ 20 ^,^ 24 ^,^ 26 ^,^ 28 ^,^ 31 ^,^ 34 ^,^ 36 ^-^ 37 ^)^. Another study presented only the process of selection of indicators^(^ 25 ^)^. The last two studies were about an implementation pilot of MHIs^(^ 30 ^)^ and a discussion of MHIs for implementation^(^ 40 ^)^.

*Studies related to HICs.* A study conducted from 1980 to 2000 in a group of 16 countries belonging to the WHO, OECD; Eurostat and IMS showed the use of macro indicators. However, it showed that the official resources for European countries indicate a lack of efficient institutional information and the need to improve the quality of MH services in European countries^(^ 19 ^)^.

Another study pointed out the profile of 8 countries in Europe, which have programs to monitor the quality of MH care implemented at their system level. Moreover, Italy and Germany are in the process of developing and implementing such programs, while in Portugal a Quality Monitoring Program in Mental Health Care (QMP-MHC) started to be implemented in 2016^(^ 21 ^)^. Currently, in Portugal, only general indicators implemented for healthcare use monitoring. Specific quality monitoring in mental healthcare is to be implemented. All countries, except the Netherlands and France, use administrative data as the main source of indicators. This shows the relevance of reliable available databases, such as those provided by claims data or health care use records, to facilitate the implementation of indicator-based quality assurance. Indicators that are constructed from administrative data are more likely to measure what is easier to measure, rather than what is relevant. Denmark, England, Germany, Holland, and Portugal also make use of reported clinical data, while the Netherlands only uses clinical or patient data. Evidence shows that the best results in quality monitoring systems are obtained if monitoring and feedback methodologies are used^(^ 21 ^)^.

The Organization for Economic Co-operation and Development (OECD) conducted a study between June and December of 2014 among its 37 member countries^(^ 22 ^)^. The objective of this particular study was to explore the reporting and use of OECD Health Care Quality Indicators (HCQI) in its member-states. The reports from these countries were most frequently focused on specific diseases and attention systems/indicators in the sector. The only indicators specific to the care of mental disorders in the study were unplanned hospital readmissions for mental disorders, unplanned schizophrenia re-admissions (same or different hospital), unplanned bipolar disorder re-admissions (same or different hospital) and excess of mortality due to mental disorders (schizophrenia/bipolar disorders). None of the reports from the countries contained responses for all four of the indicators proposed by the study, and only Belgium and Canada referred to the treatment of mental disorders. MH care and patient care with indicators of outpatient care were reported with less presence. The least mentioned indicator was “excess mortality due to mental disorders”^(^ 22 ^)^.

In a more specific study for HICs, the Italian experience in the use of clinical indicators is uneven, although Italy’s psychiatric reform in 1978 and recent legislation have delegated responsibility for planning, coordinating and delivering MH care to regions^(^ 26 ^)^. After 30 years of psychiatric reform, inequalities still remain in terms of resources and provision of services and in terms of information technology and the use of information systems. Still, in HICs, we have the Netherlands that sought to develop performance indicators seeking to improve the MH system. However, the results of this study focused on a pilot implementation of MHIs^(^ 30 ^)^. In Norway, a study published in 2004 aimed to develop a set of quality indicators to detect the impact on the quality of MH services. Focus group participants emphasize that the local context may have implications for the interpretation of indicator data and pointed out the difficulties in reaching the numerator and denominator and in defining the quality indicators of MH services. Thus, the study did not inform the implementation and use of the selected quality indicators^(^ 28 ^)^.

*Studies related to LMICs.* The first study in this group showed that more than 85% of the world’s population lives in 153 LMICs, of which 20-30% do not have MH policies, programs, and legislation. Within the WHO regions, 80% of the 191 countries had an MH policy or program and 70% had MH legislation. The analyzation between regions shows important differences, for example, 92% of the countries belonging to the European region have an MH policy, program and/or legislation. However, only 55% of the Eastern Mediterranean Region (EMR) countries have an MH policy, program and/or legislation^(^ 33 ^)^.

The EMR group of countries has 11 LMICS countries that do not have an MH policy, program and/or legislation. In the Africa and Southeast Asia regions, a total of 70% and 50% of their countries, respectively, spend less than 1% of their health budget on MH care. While 60% of the European countries spend more than 5% of their health budget on MH care. Only three African countries reported spending more than 5% of their MH financial resources^(^ 33 ^)^.

Another study finding is that the treatment gap for people with MH problems in LMICs is marked by the number of people who need care and those who receive such care. Moreover, a recent study aimed to improve MH outcomes in environments from six LMICs - Ethiopia, India, Nepal, Nigeria, South Africa and Uganda - in which they seek evidence and capacity to improve the health system, including the development, use and monitoring of indicators^(^ 36 ^,^ 41 ^)^.

Through a 10-year study, it was estimated that an additional investment of up to USD 20 *per* person/year would be needed for low-income countries, and of up to USD 30 for middle-income countries, which would result in a target expense of USD 2 and USD 3-4 *per* person, respectively. Compared to other investments, for example, the estimated total costs of increasing the neonatal health care package to 90% coverage was estimated at USD 5-10 *per capita*. Meanwhile, the cost of universal provision access to basic health services has been estimated at more than USD 30 *per* person *per* year^(^ 32 ^)^.

Another study showed that 22 Member States in the EMR have independent MH policies that have been updated over the past 10 years. Legislation needs to be reviewed among MH policies by international human rights instruments and indicated that they are partially implemented^(^ 35 ^)^. Countries from the region have the government as its main funding source (77%). However, in the remaining countries, the main funding source can be households (2 countries), non-government organizations (1 country) or unknown (2 countries did not report)^(^ 35 ^)^.

## Discussion

In all selected studies, the authors point to the relevance of using MHIs. According to the WHO guidelines in their Action Plan, several countries have attempted to define an appropriate set of indicators in the practice of MH services^(^ 1 ^,^ 35 ^)^. However, the results shown by MHIs have different uses for management, policy and service improvement^(^ 22 ^,^ 35 ^)^. Also, some countries are involved in the discussion process and in gathering the necessary indicators^(^ 21 ^,^ 33 ^)^. Nevertheless, some initiatives for the implementation of MHIs are in the process of partial implementation or in the implementation of pilot projects, suggesting that the effectiveness of these indicators is still unknown^(^ 26 ^)^. In LMICs countries, the research studies on MHIs were performed with many difficulties due to: lack of basic MH services; financial resources; legislation and political interest; MH management guidelines; and MH data integration systems^(^ 36 ^,^ 41 ^-^ 42 ^)^.

In the last decade, global MH has emerged as an important area of expression and research with the need for the development of MH services in middle-income countries. However, when comparing the health financial resources employed in MH, it is a mistake to believe that improved MH care is only needed in poorer countries. The overall understanding of health should be about improving MH everywhere, including HICs. Although these countries have state-planned systems, they create inefficient MH services and make decisions that do not adequately involve those who use the services, making them inaccessible and indifferent^(^ 43 ^)^.

Outcome quality indicators are only occasionally used to analyze MH services, and that is because most jurisdictions do not have clinical data systems to meaningfully incorporate indicators among MH providers. Nevertheless, the effectiveness of the health services remains unknown^(^ 44 ^)^.

A project in France used patient-reported experience to measure the quality of admissible MH care for adult patients with MH disorders. These measures converged on the availability, diversity, and capacity of MH care resources but they do not include “what matters to patients”. Other initiatives have been suggested to represent patients’ views, such as the patient outcome information system. This work has been of great interest in France, where they have been reporting significant regional disparities in the MH system, without significant change, in recent decades^(^ 45 ^)^.

The process of implementing MHIs is a difficult task even for European countries. Portugal can be used as an example, with its National Health Plan being carried out throughout the 2017-2020 period; however, MHIs have not been implemented using proposals made by the Working Group. However, these proposed measures do not have an integrated strategy for the promotion and prevention of MH. According to the report, these changes will only be possible if they are developed within the framework of coordination teams that have the capacity for action at the inter-sectorial level^(^ 46 ^)^.

Moreover, this is a particular concern in LMICs, where more than 70% of mental illnesses occur. Poor access to MH services has been highlighted, ranging from less than 50% to less than 10% in many countries. In LMICs, the difference between those in need of treatment and resource availability is almost 90%^(^ 47 ^)^.

In the case of Brazil, India and South Africa, a study points out that the financial resources earmarked are very scarce and that they have inadequate human resources and infrastructure for MH. In the study, India reported that the national program was implemented only in small pockets (locations), after two decades of its acceptance, due to a lack of budget allocation for national use. In the case of South Africa, the study suggested that MH policy and legislation do not automatically translate into adequate MH services if they are not clearly defined by a comprehensive national program^(^ 33 ^)^. In Brazil, important advances occurred in the 1980s, such as changes in politics and strengthening of the workforce. This resulted in a major reform of the MH system; changes in care delivery with the creation of new Community Psychosocial Centers (CAPS); and specialized MH services to provide outpatient care^(^ 12 ^,^ 48 ^)^. However, until recently, the only specific indicator for specialized MH service evaluation used in Brazil was the number of CAPS, while other indicators for MH aspects of primary care were also computed by the Program for Improving Access and Quality in Primary Care (PMaQ)^(^ 42 ^)^. Nowadays, Brazil has been utilizing an indicator defined through the Inter-Federative Pact 2017-2021, which refers to an action set of systematic matrices performed by the CAPS in partnership with primary care teams^(^ 49 ^)^.

Political reforms in the country and the commitment of health professionals to provide care in the primary health care system were the main facilitators for their success. However, many obstacles need to be overcome, concerns such as unequal distribution and coverage of community services in all regions, as well as the government’s failure to increase resources for MH care remain major challenges. The effect of changes in the policy of providing MH care throughout Brazil needs to be evaluated regularly to improve and tune the system^(^ 12 ^,^ 42 ^)^. Therefore, the existing Brazilian indicators in MH cannot support consistent assessments of the model adopted in recent years and it is necessary to create indicators that cover all MH aspects of the population.

Some obstacles are pointed out in the literature on the use of indicators and difficulties in their implementation, professionals and managers view these indicators as threats, due to their unreliability, and as tools to penalize poor performance, including fear of area-based financial penalties and the integrative of professional control. Other issues indicate some distrust in the use of government-associated evaluators, lack of diagnoses defined in graphs, and difficulties to define intervention limits. Important financial issues emerge when discussing the use of indicators such as lack of equipment and internet access. The indicator generation is a costly task that requires intensive work and outside staff, this includes the need for computer training and increased workload as well as predefined routines and protocols.

Some other possible issues are as follows: information services may not be recorded in medical records or are difficult to find; lack of clarity and agreement on data entry; lack of time for planning professionals; and lack of staff approach to adjust to the changes of using indicators^(^ 50 ^)^.

For MH to find strong and widely agreed upon indicators of health and mental illness, there must be an improvement in the quality of care and in the attention in health services. Our results reinforce the importance of the indicators in all phases of the mental health-disease process and, therefore, to the entire network of health services. With these results, it seems important that specialized psychiatric prevention services implement health promotion programs specifically targeting psychiatric patients^(^ 20 ^,^ 51 ^)^.

A limitation of this review is that it should involve a detailed search of the normative documents, we only consulted and compared data found in scientific papers, there were difficulties in finding data sources with indicator information, calculation method and information systems of each country. A complementary work to this study is being carried out to obtain MHIs that suit Brazilian Health Information Systems and that can be implemented using the available data^(^ 52 ^)^.

A strength to this review is that it shows that, by extracting knowledge on the use of a set of MHIs, it is possible to understand the implementation phase of indicators in different regions of the world and to compare them with each other. The MHI initiatives of several countries were in the implementation or development phase and this reflects the lack of maturity and consistency in the application of MHIs in most countries. Based on the MHIs evaluated in this study, we observe that there is no consensus regarding their use for MH management. In addition, we confirm the previous findings that there is no consensus on the definition, method of calculation, and management level of the indicators that are used.

## Conclusion

The main findings of this review show that it is unusual to find a descriptive comparison of quality-monitoring programs at the system level in the technical-scientific literature related to MH. This occurs not only because such systems are rare or in development, but also because most programs are managed by national public agencies whose purpose is not to publish results in the scientific literature. Global initiatives are underway and seek to expand MH services to address the treatment and care gap.

Indicators are important information tools to map advances, setbacks or stagnation in different aspects and sectors of society. An analysis of indicators by the public and private health financing systems has not been found even in countries that have both systems. Indicators for each system may present relevant differences for analysis. In this sense, this review contributes to this scenario by extracting knowledge and establishing an updated framework on the use of MHI for care and management.

When we mention the importance of using indicators, we point out that they are intended to help individuals, understand the performance of community health services, and provide information easily and conveniently to reflect changes in time and to assess appropriate service performance to effectively meet customer needs. In developing indicators, patients and the integrative of indicators seek to understand MH problems, including mental disorders; resources, skilled labor, facilities and finances; MH services; risks; protective factors, and so on. They enhance MH status, factors, system, and quality of MH services to include prevention, treatment, ongoing management, and early interventions. Developed countries invest in MHIs systematically based on theoretical foundations, such as national projects, that not only assess the population’s MH status and monitor trends, but also provide scientific research to policymakers as well as monitor processes and policy outcomes. Indicators are useful screening tools for potential problems in preventive and primary care. They also determine if there is a quality problem and the need for further analysis on a given topic.

This scenario suggests the need for a set of indicators to be standardized by the WHO, serving as an evidence-based guide to best practices available. Also, we have suggested more flexibility and adaptability, taking into account the reality of each country. To achieve this goal, the development of indicators must be carried out by professionals within the entire health service network.

## References

[1] World Health Organization (2013). Mental health action plan 2013 - 2020.

[2] Lima IB, Yamada DB, Yoshiura VT, Lance RC, Rodrigues LML, Vinci ALT (2018). Proposal for selection of mental health indicators in the management of health networks: from heuristic to process modeling. Procedia Comput Sci.

[3] Anderson KK, Fuhrer R, Schmitz N, Malla AK (2012). Determinants of negative pathways to care and their impact on service disengagement in first-episode psychosis. Soc Psychiatry Psychiatr Epidemiol.

[4] World Health Organization (2015). Mental Health Atlas 2014.

[5] World Health Organization (2018). Mental Health Atlas 2017.

[6] Vigo D, Thornicroft G, Atun R (2016). Estimating the true global burden of mental illness. Lancet Psychiatry.

[7] Bauer MS (2002). A Review of Quantitative Studies of Adherence to Mental Health Clinical Practice Guidelines. Harvard Rev Psychiatry.

[8] Docherty M, Shaw K, Goulding L, Parke H, Eassom E, Ali F (2017). Evidence-based guideline implementation in low and middle income countries: lessons for mental health care. Int J Ment Health Syst.

[9] Kilbourne AM, Keyser D, Pincus HA (2010). Challenges and Opportunities in Measuring the Quality of Mental Health Care. Can J Psychiatry.

[10] Girlanda F, Fiedler I, Ay E, Barbui C, Koesters M (2013). Guideline implementation strategies for specialist mental healthcare. Curr Opin Psychiatry.

[11] Weinmann S, Koesters M, Becker T (2007). Effects of implementation of psychiatric guidelines on provider performance and patient outcome: systematic review. Acta Psychiatr Scand.

[12] Dantas CDR, Oda AMGR (2014). Cartografia das pesquisas avaliativas de serviços de saúde mental no Brasil (2004-2013). Physis Rev Saude Colet.

[13] The Joanna Briggs Institute (2015). The Joanna Briggs Institute Reviewers' Manual 2015.

[14] Dal K, Mendes S, Cristina de Campos R, Silveira P, Galvão CM (2008). Integrative Literature Review: A research method to incorporate evidence in health care and nursing. Texto Contexto Enferm.

[15] Arksey H, Omalley L (2005). Scoping studies: towards a methodological framework. Int J Soc Res Methodol.

[16] Medical Subject Headings U.S. National Library of Medicine; National Institutes of Health.

[17] Butler A, Hall H, Copnell B (2016). A Guide to Writing a Qualitative Systematic Review Protocol to Enhance Evidence-Based Practice in Nursing and Health Care. Worldviews Evid Based Nurs.

[18] Moher D (2009). Preferred Reporting Items for Systematic Reviews and Meta-Analyses: The PRISMA Statement. Ann Intern Med.

[19] Carta MG, Kovess V, Hardoy MC, Brugha T, Fryers T, Lehtinen V (2004). Psychosocial wellbeing and psychiatric care in the European Communities: analysis of macro indicators. Soc Psychiatry Psychiatr Epidemiol.

[20] Amaddeo F, Tansella M (2011). New perspectives of mental health service research. Epidemiol Psychiatr Sci.

[21] Bramesfeld A, Amaddeo F, Caldas-De-Almeida J, Cardoso G, Depaigne-Loth A, Derenne R (2016). Monitoring mental healthcare on a system level: Country profiles and status from EU countries. Health Policy.

[22] Rotar AM, Berg MJVD, Kringos DS, Klazinga NS (2016). Reporting and use of the OECD Health Care Quality Indicators at national and regional level in 15 countries. Int J Qual Health Care.

[23] Thomson R (2004). UK Quality Indicator Project(R) (UK QIP) and the UK independent health care sector: a new development. International J Qual Health Care.

[24] Kinkorová J, Topolcan O (2012). Overview of healthcare system in the Czech Republic. EPMA J.

[25] Han H, Ahn DH, Song J, Hwang TY, Roh S (2012). Development of Mental Health Indicators in Korea. Psychiatry Invest.

[26] Lora A (2012). Call for information, call for quality in mental health care. Epidemiol Psychiatr Sci.

[27] Ala-Nikkola T, Pirkola S, Kontio R, Joffe G, Pankakoski M, Malin M (2014). Size Matters - Determinants of Modern, Community-Oriented Mental Health Services. Int J Env Res Pub Health.

[28] Hartveit M, Vanhaecht K, Thorsen O, Biringer E, Haug K, Aslaksen A (2017). Quality indicators for the referral process from primary to specialised mental health care: an explorative study in accordance with the RAND appropriateness method. BMC Health Serv Res.

[29] Gaebel W, Janssen B, Zielasek J (2009). Mental health quality, outcome measurement, and improvement in Germany. Curr Opin Psychiatry.

[30] Lauriks S, Wit MASD, Buster MCA, Arah OA, Klazinga NS (2014). Composing a Core Set of Performance Indicators for Public Mental Health Care: A Modified Delphi Procedure. Admin Policy Ment Health.

[31] Dixon A, Mcdaid D, Knapp M, Curran C (2006). Financing mental health services in low- and middle-income countries. Health Policy Plan.

[32] Lancet Global Mental Health Group (2007). Scale up services for mental disorders: a call for action. Lancet.

[33] Jacob K, Sharan P, Mirza I, Garrido-Cumbrera M, Seedat S, Mari J (2007). Mental health systems in countries: where are we now?. Lancet.

[34] Cohen A, Eaton J, Radtke B, George C, Manuel B, Silva MD (2011). Three models of community mental health services in low-income countries. Int J Ment Health Syst.

[35] Chew Z, Saeed K (2015). Situational analysis: preliminary regional review of the Mental Health Atlas 2014. Eastern Mediterr Health J.

[36] Jordans MJD, Chisholm D, Semrau M, Upadhaya N, Abdulmalik J, Ahuja S (2016). Indicators for routine monitoring of effective mental healthcare coverage in low- and middle-income settings: a Delphi study. Health Pol Plan.

[37] Hanlon C, Semrau M, Alem A, Abayneh S, Abdulmalik J, Docrat S (2017). Evaluating capacity-building for mental health system strengthening in low- and middle-income countries for service users and caregivers, service planners and researchers. Epidemiol Psychiatr Sci.

[38] Lund C, Kleintjes S, Kakuma R, Flisher AJ (2009). Public sector mental health systems in South Africa: inter-provincial comparisons and policy implications. Soc Psychiatry Psychiatr Epidemiol.

[39] Hernandez LJ (2003). Evaluación de resultados e impactos de un Modelo de Salud Mental Basado en la Comunidad en localidades de Bogotá, DC. Rev Salud Pública.

[40] Ahuja S, Gronholm PC, Shidhaye R, Jordans M, Thornicroft G (2018). Development of mental health indicators at the district level in Madhya Pradesh, India: mixed methods study. BMC Health Serv Res.

[41] Upadhaya N, Jordans MJD, Abdulmalik J, Ahuja S, Alem A, Hanlon C (2016). Information systems for mental health in six low and middle income countries: cross country situation analysis. Int J Ment Health Syst.

[42] Mendes MFDM, Rocha CMFD (2016). Avaliação Em Saúde Mental: Uma Análise de Políticas Nacionais e Internacionais. Saúde Redes.

[43] Sashidharan SP, White R, Mezzina R, Jansen S, Gishoma D (2016). Global mental health in high-income countries. Br J Psychiatry.

[44] Perlman CM, Hirdes JP, Barbaree H, Fries BE, Mckillop I, Morris JN (2013). Development of mental health quality indicators (MHQIs) for inpatient psychiatry based on the interRAI mental health assessment. BMC Health Serv Res.

[45] Fernandes S, Fond G, Zendjidjian X, Michel P, Baumstarck K, Lancon C (2019). The Patient-Reported Experience Measure for Improving quality of care in Mental health (PREMIUM) project in France: study protocol for the development and implementation strategy. Patient Prefer Adherence.

[46] Xavier M, Paixão I, Mateus P, Goldschmidt T, Pires P, Narigão M (2017). Relatório da Avaliação do Plano Nacional de Saúde Mental 2007-2016 e propostas prioritárias para a extensão a 2020..

[47] Alloh FT, Regmi P, Onche I, Teijlingen EV, Trenoweth S (2018). Mental Health in low-and middle income countries (LMICs): Going beyond the need for funding. Health Prospect.

[48] Ministério da Saúde (BR) (2011). Portaria No-3.088, de 23 de dezembro de 2011. Institui a Rede de Atenção Psicossocial para pessoas com sofrimento ou transtorno mental e com necessidades decorrentes do uso de crack, álcool e outras drogas, no âmbito do Sistema Único de Saúde. Diário Oficial da União.

[49] Ministério da Saúde (BR) (2017). Pactuação Interfederativa 2017-2021: Fichas de Indicadores.

[50] Addington D, Bs MB, Kyle T, Desai S, Wang J (2010). Research Facilitators and barriers to implementing quality measurement in primary mental health care: systematic review. Can Fam Physician.

[51] Mai Q, Holman CDJ, Sanfilippo FM, Emery JD (2011). The impact of mental illness on potentially preventable hospitalisations: a population-based cohort study. BMC Psychiatry.

[52] Vinci ALT, Lima IB, Rijo RPCL, Alves D (2019). Mental Health Indicators Set for Management and Evaluation of a Mental Healthcare Network: Implementation Feasibility Analysis, to Administration and Policy in Mental Health and Mental Health Services Research. Admin Pol Ment Health.

